# Association between Carotid Plaque Characteristics and Cerebral White Matter Lesions: One-Year Follow-Up Study by MRI

**DOI:** 10.1371/journal.pone.0017070

**Published:** 2011-02-15

**Authors:** Robert M. Kwee, Paul A. M. Hofman, Ed H. B. M. Gronenschild, Robert J. van Oostenbrugge, Werner H. Mess, Johannes W. M. ter. Berg, Cees L. Franke, Arthur G. G. C. Korten, Bé J. Meems, Jos M. A. van Engelshoven, Joachim E. Wildberger, M. Eline Kooi

**Affiliations:** 1 Department of Radiology, Maastricht University Medical Center, Maastricht, The Netherlands; 2 Cardiovascular Research Institute Maastricht, Maastricht University Medical Center, Maastricht, The Netherlands; 3 Department of Psychiatry, Maastricht University Medical Center, Maastricht, The Netherlands; 4 Department of Neuropsychology, Maastricht University Medical Center, Maastricht, The Netherlands; 5 Department of Neurology, Maastricht University Medical Center, Maastricht, The Netherlands; 6 Department of Clinical Neurophysiology, Maastricht University Medical Center, Maastricht, The Netherlands; 7 Department of Neurology, Orbis Medical Center, Sittard, The Netherlands; 8 Department of Neurology, Atrium Medical Center, Heerlen, The Netherlands; 9 Department of Neurology, Laurentius Hospital, Roermond, The Netherlands; 10 Department of Neurology, Vie Curi Medical Center, Venlo, The Netherlands; Julius-Maximilians-Universität Würzburg, Germany

## Abstract

**Objective:**

To prospectively assess the relation between carotid plaque characteristics and the development of new cerebral white matter lesions (WMLs) at MRI.

**Methods:**

Fifty TIA/stroke patients with ipsilateral 30–69% carotid stenosis underwent MRI of the plaque at baseline. Total plaque volume and markers of vulnerability to thromboembolism (lipid-rich necrotic core [LRNC] volume, fibrous cap [FC] status, and presence of intraplaque hemorrhage [IPH]) were assessed. All patients also underwent brain MRI at baseline and after one year. Ipsilateral cerebral WMLs were quantified with a semiautomatic method.

**Results:**

Mean WML volume significantly increased over a one-year period (6.52 vs. 6.97 mm^3^, *P* = 0.005). WML volume at baseline and WML progression did not significantly differ (*P*>0.05) between patients with 30–49% and patients with 50–69% stenosis. There was a significant correlation between total plaque volume and baseline ipsilateral WML volume (Spearman ρ = 0.393, *P* = 0.005). There was no significant correlation between total plaque volume and ipsilateral WML progression. There were no significant associations between LRNC volume and WML volume at baseline and WML progression. WML volume at baseline and WML progression did not significantly differ between patients with a thick and intact FC and patients with a thin and/or ruptured FC. WML volume at baseline and WML progression also did not significantly differ between patients with and without IPH.

**Conclusion:**

The results of this study indicate that carotid plaque burden is significantly associated with WML severity, but that there is no causal relationship between carotid plaque vulnerability and the occurrence of WMLs.

## Introduction

Carotid atherosclerosis is an important cause of ischemic stroke. The pathogenesis of ischemic stroke due to carotid atherosclerosis can be ascribed to cerebral embolism from carotid plaque and/or hypoperfusion due to stenosis [1]. Large epidemiological studies showed that there also is a relation between the presence and amount of carotid atherosclerosis and white matter lesions (WMLs) [2], [3], which are frequently detected by magnetic resonance imaging (MRI) in the aging brain[4]. The presence and severity of WMLs have substantial clinical relevance, because they are associated with cognitive decline [5], dementia [6], and impaired mobility [7]. Moreover, it has been shown that WMLs are a strong independent predictor of stroke [8], [9] and global functional decline [10]. It is unclear yet whether the relation between carotid atherosclerosis and WMLs is indirect via shared risk factors or causative in nature. To our knowledge, it has not been investigated yet whether the presence of certain carotid plaque features precedes the development of new WMLs. Therefore, the purpose of the present study was to prospectively assess the relation between carotid plaque characteristics and cerebral WML progression at MRI.

## Materials and Methods

### Patients

Patients with recent (<3 months) amaurosis fugax, TIA, or minor stroke and an ipsilateral carotid plaque causing 30–69% stenosis, as determined by ultrasonography, were eligible for inclusion. Exclusion criteria were: atrial fibrillation or other cardiogenic cause of TIA/stroke, standard contraindications for MRI, and a renal clearance <30 ml/min/1.73 m^2^. Participating patients underwent baseline and follow-up MRI after one year (see MRI protocol below). This study was approved by the institutional review board of our hospital. All patients gave written informed consent.

### Clinical parameters

For each patient age and sex were recorded. Hypertension was defined as a systolic blood pressure ≥140 mm Hg and/or a diastolic blood pressure ≥90 mm Hg, or treatment with antihypertensive medication [11]. Diabetes mellitus was defined as reported use of medication for diabetes mellitus or fasting plasma glucose level ≥126 mg/dl [12]. Patients were categorized into current, former and never smokers.

### MRI protocol

A baseline MRI scan of the carotid plaque ipsilateral to the presumed side of ischemia was obtained using a dedicated 47 mm-diameter surface coil, as described previously [13]. Five MR pulse sequences were acquired: (1) 3D T1-weighted TFE: TR/TI/TE 10.3/900/4.4 ms, flip angle 15°, inversion prepulse, shot interval time 3000 msec, TFE factor 163, slice thickness 3.0 mm; (2) 3D TOF: TR/TE 23/3.9 ms, flip angle 25°, slice thickness 3.0 mm; (3) Multislice T2-weighted TSE: TR/TE 2 heartbeats/50 ms, echo train length 8, slice thickness/gap 2.5/0.5 mm; (4/5) Pre- and post-contrast 2D T1-weighted TSE (double inversion-recovery): TR/TI/TE 1 heartbeat/heart rate dependent/18 ms, echo train length 9, slice thickness/gap 2.5/0.5 mm. The post-contrast T1-weighted TSE sequence was obtained 7-8 minutes after injection of 0.1 mmol/kg body weight of gadopentetate dimeglumine (Magnevist, Bayer Schering Pharma AG, Berlin, Germany). T1 was adjusted according to heart rate and postcontrast T1 relaxation time of blood. For all sequences the FOV was 100×80 mm, with a matrix size of 256×205 (in-plane resolution, 0.39×0.39 mm), except for the T1-weighted TFE sequence (FOV 100×80 mm, matrix size 256×163; in-plane resolution 0.39×0.49 mm).

In addition, brain MRI scans at baseline and after one year follow-up were obtained using a standard quadrature head coil, as described previously [14]. Two MR pulse sequences were acquired: (1) T2-weighted TSE: TR/TE 4820/100 ms, echo train length 12, slice thickness/gap 5.0/0.5 mm; (2) fluid-attenuated inversion recovery (FLAIR): TR/TI/TE 8000/2000/120 ms, echo train length 23, slice thickness/gap 5.0/0.5 mm. For both sequences the FOV was 230×230 mm, with a matrix size of 512×512 mm (in-plane resolution 0.45×0.45 mm).

All MRI examinations were performed on a 1.5-T whole-body scanner (Intera 11.1.4.4, Philips Healthcare, Best, the Netherlands).

### MR image interpretation

**Figure 1:**
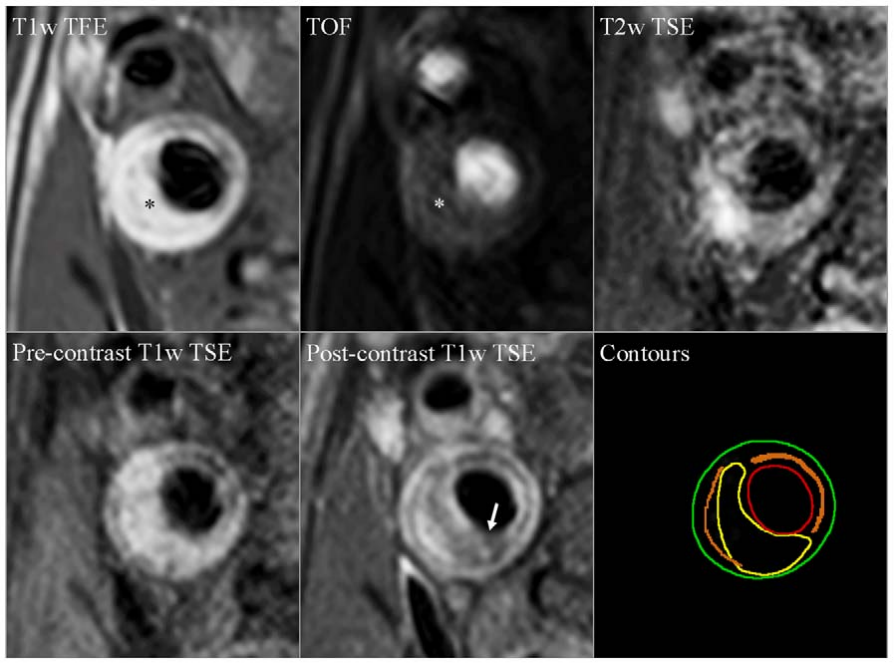
Co-registered T1-weighted TFE, TOF, T2-weighted TSE, pre- and post-contrast T1-weighted TSE images of a transverse section of a plaque in the internal carotid artery. The right bottom panel displays the plaque components: red = lumen; green = outer vessel wall; yellow = LRNC; orange = calcifications; remaining vessel wall area = fibrous tissue. IPH was scored as being present (asterisk in T1-weighted TFE and TOF images) and the FC was designated as thin and/or ruptured (arrow in post-contrast T1-weighted TSE image).

MR images of the plaque were evaluated by one investigator (R.M.K.), blinded to all patient information, including brain MR images, using dedicated vessel wall analysis software (VesselMASS, Department of Radiology, Leiden University Medical Center, The Netherlands). For each plaque, total plaque volume, lipid-rich necrotic core (LRNC) volume, fibrous cap (FC) status, and presence of intraplaque hemorrhage (IPH) were assessed (all are histopathologic features of vulnerability to thromboembolism), as described previously [13], [14] (Figure 1 gives an example). Previously, we showed in a comparable group of consecutive patients that intra- and interobserver agreement for assessment of total plaque volume, LRNC volume, and FC status assessment were (very) good (ICC = 0.71–0.92, and κ =  0.60–0.98) [13], [14], while intra- and interobserver agreement for the detection of IPH were very good to excellent (κ = 0.86–1.00) [13].

**Figure 2:**
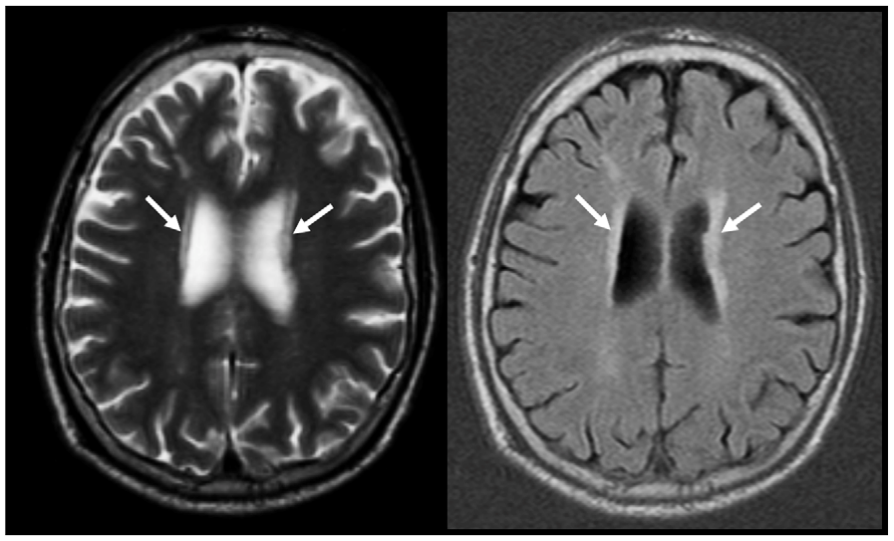
Transverse T2-weighted TSE and fluid-attenuated inversion recovery images showing WMLs, mainly located adjacent to the ventricles (arrows).

MR images of the brain (Figure 2 gives an example) were evaluated ≥12 months after carotid plaque analysis by one investigator (R.M.K.) who was blinded to all patient information, the time point at which the MRI examination was performed, and the results of carotid plaque analysis. Three months after the first reading, one observer (R.M.K.) re-evaluated all MR brain images of 20 consecutive patients, blinded to the results of the first reading session, to assess intraobserver variability. In addition, an experienced neuroradiologist (P.A.M.H.) also prospectively analyzed MR brain images of 20 consecutive patients independently to assess interobserver variability. Intra- and interobserver agreement for assessment of WML volume were excellent ICCs of 0.99). Total, deep, and periventricular WML volumes of the ipsilateral cerebral hemisphere were assessed using dedicated semi-automated software (GIANT, E.H.B.M.G., Department of Psychiatry and Neuropsychology, Maastricht University Medical Center, the Netherlands) [15].

### Statistical analysis

Statistical analysis was performed using SPSS version 11.5 (SPSS Inc, Chicago, USA). Differences between WML volume at baseline and after one-year follow-up were assessed by the Wilcoxon signed-rank test. Associations between clinical parameters and WML volume at baseline and WML volume progression were assessed by Spearman rank correlation tests. Associations between total plaque volume, carotid plaque LRNC volume and ipsilateral WML volume at baseline and WML volume progression were also assessed by Spearman rank correlation tests and a scatter plot was generated. Differences in ipsilateral WML volume at baseline and WML volume progression between patients with different stenosis grade, patients with different FC status, and between those with and without carotid IPH, were evaluated using the Mann-Whitney U test and by generating Box-and-Whisker plots. In addition, an analysis was performed for the relation between carotid plaque characteristics and deep and periventricular WMLs separately, and for the relation between carotid plaque characteristics and for left and right sided plaques separately. Level of significance was set at 0.05.

## Results

Fifty Caucasian patients undergoing baseline and follow-up MRI after one year (see MRI protocol below), were included. Patient characteristics are presented in Table 1. Mean time interval between last symptoms and baseline MRI examination was 30±19 days. According to clinical guidelines, all 50 patients were prescribed aspirin and dipyridamole. Forty-seven patients were prescribed statins. No carotid surgical interventions were performed and none of the included patients experienced a stroke during the follow-up period.

**Table 1:** Patient characteristics (n = 50).

Sex (male: female)	30∶20
Mean age (range)	67.7±9.9 (47–87) years
Patients with hypertension	42
Patients with diabetes mellitus	10
Smoking habits	
Never smokers	18
Former smokers	25
Current smokers	7
Mean total plaque volume (range)	943.3±286.0 (458.5–2000.6) mm^3^
Mean LRNC volume (range)	96.5±147.44 (0–557.2) mm^3^
FC status	
Thick and intact	29
Thin and/or ruptured	21
IPH present	16
Mean ipsilateral WML	6.52±8.17 (0.23–29.72) mm^3^
volume at baseline (range)	
Mean ipsilateral WML	6.97±8.88 (0.17–32.02) mm^3^
volume after one year (range)	

Mean ipsilateral WML volume significantly increased over a one-year period (6.52 vs. 6.97 mm^3^, *P* = 0.005). Age and hypertension were significantly associated with baseline WML volume, while age and WML volume at baseline were significantly associated with WML progression. In addition, males tended to have higher WML volume at baseline and higher WML progression than woman, albeit these findings were not statistically significant (Table 2).”

**Table 2:** Results of Spearman rank correlation analyses for associations between clinical parameters, ipsilateral WML volume at baseline and ipsilateral WML volume progression after one year. Significant results are displayed in bold.

	Ipsilateral WML volume at baseline		Ipsilateral WML volume difference after one year	
	Spearman ρ	*P*-value	Spearman ρ	*P*-value
**Age**	**0.542**	**<0.001**	**0.371**	**0.008**
**Sex**	−0.267	0.061	−0.229	0.109
**Hypertension** a	**0.329**	**0.020**	0.144	0.320
**Diabetes mellitus** b	0.010	0.943	0.159	0.269
**Smoking** c	−0.123	0.396	−0.046	0.753
**Ipsilateral WML volume at baseline**	NA	NA	**0.483**	**<0.001**

a Hypertension was defined as a systolic blood pressure ≥140 mm Hg and/or a diastolic blood pressure ≥90 mm Hg, or treatment with antihypertensive medication.

b Diabetes mellitus was defined as reported use of medication for diabetes mellitus or fasting plasma glucose level ≥126 mg/dl.

c Patients were categorized into current, former and never smokers.

NA: not applicable

**Figure 3:**
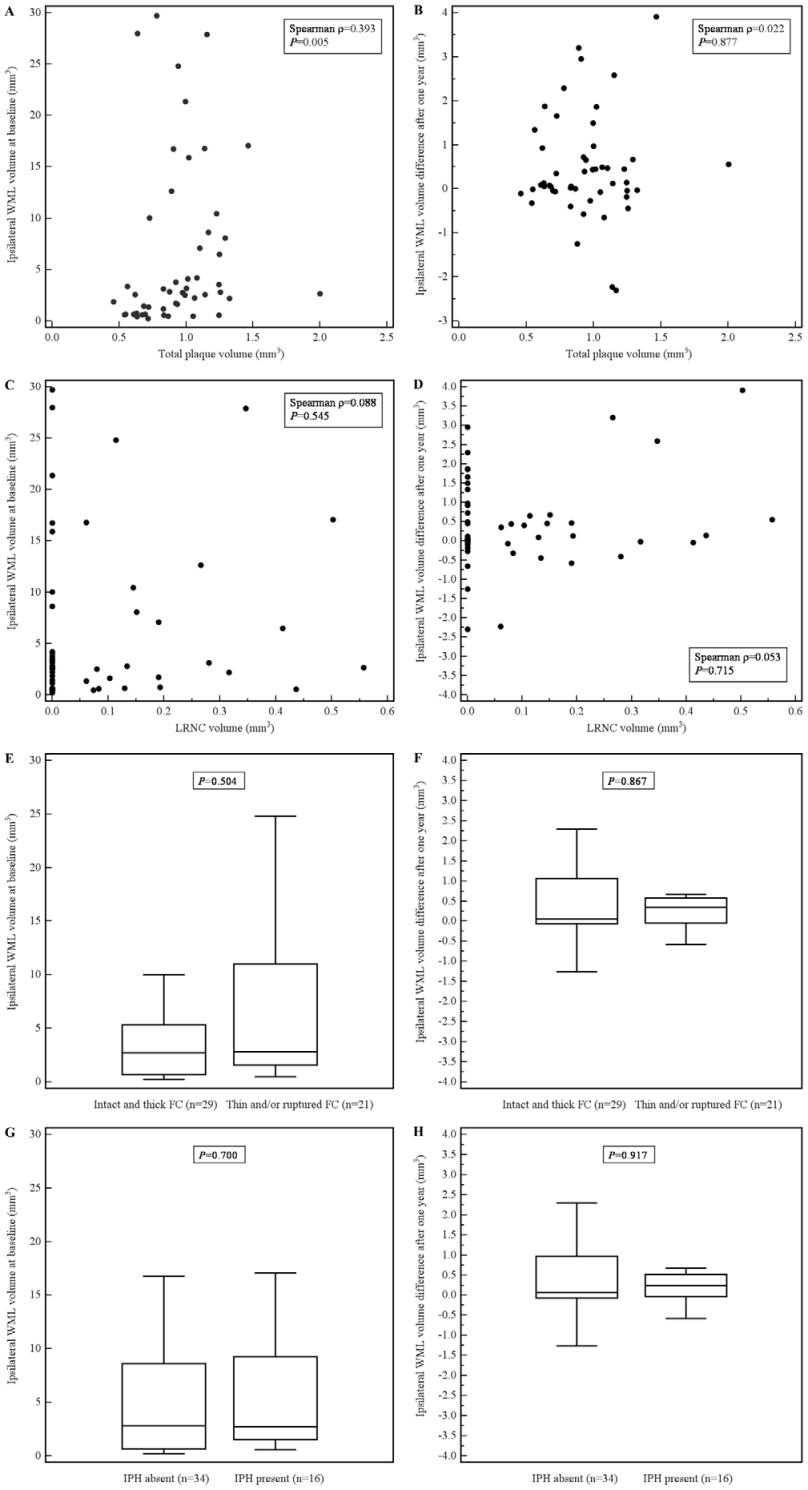
Scatter plots showing the relation between total plaque volume and ipsilateral WML volume at baseline (A) and ipsilateral WML volume difference after one year (B); and between LRNC volume and ipsilateral WML volume at baseline (C) and ipsilateral WML volume difference after one year (D). Box-and-Whisker plots showing the relation between fibrous cap status and WML volume at baseline (E) and WML volume difference after one year (F). Box-and-Whisker plots showing the relation between intraplaque hemorrhage and WML volume at baseline (G) and WML volume difference after one year (H).

Baseline ipsilateral WML volume and WML progression did not significantly differ between 31 patients with 30–49% and 19 patients with 50–69% carotid stenosis (*P* = 0.689 and *P* = 0.342, respectively). Total plaque volume and baseline ipsilateral WML volume were significantly correlated (Spearman ρ = 0.393, *P* = 0.005) (Figure 3A). Total plaque volume and WML progression were not significantly correlated (Spearman ρ = 0.022, *P* = 0.877) (Figure 3B). In addition, there was no significant association between LRNC volume and baseline ipsilateral WML volume (Spearman ρ = 0.088, *P* = 0.545) and WML progression (Spearman ρ = 0.053, *P* = 0.715) (Figure 3C–D). Baseline ipsilateral WML volume and WML progression did not significantly differ between patients with a thick and intact FC and patients with a thin and/or ruptured FC (*P* = 0.504 and *P* = 0.867, respectively) (Figure 3E–F). Baseline ipsilateral WML volume and ipsilateral WML progression also did not significantly differ between patients with and without IPH (*P* = 0.700 and *P* = 0.917, respectively) (Figure 3G–H). Total plaque volume was significantly associated with ipsilateral periventricular WML volume at baseline (Spearman ρ = 0.426, *P* = 0.002). There were no significant associations between other carotid plaque characteristics and deep and periventricular WMLs separately. In addition, total plaque volume was (borderline) significantly associated with ipsilateral WML severity at baseline both for left (n = 27) and right (n = 23) sided plaques (Spearman ρ = 0.421, *P* = 0.029 and Spearman ρ = 0.400, *P* = 0.058, respectively). There were no significant associations between other carotid plaque characteristics and WMLs at the left and right side separately. Multivariate linear regression analysis, adjusted for age and hypertension, did not show significant relations between carotid plaque features and WML severity and progression.

## Discussion

In the present longitudinal follow-up study, mean ipsilateral WML volume significantly increased in TIA/stroke patients with ipsilateral carotid stenosis over a one-year period.

We found a significant association between carotid plaque burden (measured as total plaque volume) and WML severity. However, we found no associations between MRI features reflecting vulnerability to thromboembolism and ipsilateral WML severity and progression over a one-year period.

Age and hypertension were significantly associated with ipsilateral WML severity at baseline. Age and ipsilateral WML volume at baseline were significantly associated with WML progression. Our findings are consistent with epidemiologic studies which have established that age and hypertension are associated with WML severity and progression [4], [16]–[18], and that the amount of WMLs at baseline predicts WML progression [19], [20].

We found that carotid stenosis grade is not related to ipsilateral baseline WML volume, which is in line with results of earlier cross-sectional studies investigating the relation between carotid plaque characteristics and WMLs at MRI [21]–[23].

Plaque burden was found to be associated with WML severity at baseline, which is in accordance to the results of the population-based study by Pico et al. [3], who assessed carotid plaque burden by ultrasonography.

In the present study, we found no relation between markers of vulnerability to thromboembolism and WML severity and progression. We found no association between ipsilateral LRNC volume and baseline ipsilateral WML volume, which is in accordance to the cross-sectional studies by Patterson et al. [22] and Ouhlous et al. [23]. We also found no association between the presence of IPH and baseline ipsilateral WML volume, which is in line with Patterson et al. 's findings [22], but contradicts those of Altaf et al. [21]. Altaf et al. [21] suggested that embolic events from vulnerable carotid plaques with IPH may contribute to the development of WML. However, the cross-sectional design of their study [21] precluded the authors from drawing definitive conclusions. In the present longitudinal follow-up study, we found no association between the presence of carotid IPH and progression of ipsilateral WMLs. Finally, we found no associations between LRNC volume and FC status (which are also believed to be markers of plaque vulnerability) and ipsilateral WML progression.

The pathogenesis of WMLs is not fully understood yet. A concurrence of mechanisms (ischemia, blood-brain barrier alterations, edema, loss of autoregulation, etc.) may contribute to the development of WMLs [24]. Brain hypoperfusion due to luminal narrowing by carotid atherosclerosis may be one of the causes of WMLs. The occurrence of WMLs has complex relations with cardiovascular risk factors, and an indirect link between large vessel atherosclerosis and WMLs by shared risk factors is also well possible. The findings of the present study suggest that there is no causal relationship between carotid plaque vulnerability, i.e. thromboembolism from carotid plaques, and the occurrence of WMLs.

Our study has several limitations. First, this was an exploratory study. Although no noticeable effects in the distribution of WML volumes at baseline and WML volume differences after one year for LRNC volume, FC status, and IPH were observed, our findings should be confirmed by an independent study. Second, we only performed MR imaging of plaques ipsilateral to the presumed side of ischemia, which are in general more vulnerable than clinically asymptomatic carotid plaques [25]. Because of time constraints and patient inconvenience, we did not obtain MR images of contralateral asymptomatic plaques. The association between characteristics of clinically asymptomatic plaques and WML progression remains to be investigated. Third, for all patients we assessed WML volumes of the ipsilateral cerebral hemisphere, which may in fact not have been the true internal carotid artery distribution territory for each individual patient. This may only be determined by using dedicated techniques, such as selective arterial spin labeling. Fourth, our follow-up period comprised one year, the period in which carotid plaque vulnerability may be highest after the initial event [26]. Longer follow-up might have detected associations between carotid plaque characteristics and WML progression. However, this may be unlikely because in the present study we also found no association between plaque characteristics and WML severity at baseline. Finally, all patients were prescribed antiplatelet therapy and most patients were prescribed statins for secondary stroke prevention. The use of these medications may have influenced the natural history of carotid plaques, WMLs, and their possible interaction mechanisms. For instance, it has been shown that the use of statins is negatively associated with complicated plaque features [27], whereas the use of antiplatelet therapy can decrease WML progression [28]. Future studies should ideally be conducted in patients who are not using these medications. However, due to ethical reasons, this can only be done in an asymptomatic population.

In conclusion, in TIA/stroke patients with carotid stenosis, we found an association between carotid plaque burden and WML severity. We found no associations between LRNC volume, FC status, and the presence of IPH ipsilateral WML severity and progression over a one-year period, suggesting that there is no causal relationship between carotid plaque vulnerability and the occurrence of WMLs.
