# Correction: Association between Carotid Plaque Characteristics and Cerebral White Matter Lesions: One-Year Follow-Up Study by MRI

**DOI:** 10.1371/journal.pone.0103924

**Published:** 2014-07-30

**Authors:** The PLOS ONE Staff

In Table 1, “6.52 ± 8.17 (0.23−29.72) mm3” should be“6.52 ± 8.17 (0.23−29.72) ml” and “6.97 ± 8.88 (0.17−32.02) mm3” should be “6.97 ± 8.88 (0.17−32.02) ml”. Please note that in Table 1, total plaque volume and mean LRNC are correctly expressed in mm3. The authors have provided a corrected version of Table 1 here.

Throughout the article, Figure 3A-D (x and y-axis) and 3E-H (y-axis) “mm3” should be “ml”. Please view the corrected version of Figure 3 below.

**Table 1:** Patient characteristics (n  =  50).

Sex (male : female)	30 : 20
Mean age (range)	67.7±9.9 (47-87) years
Patients with hypertension	42
Patients with diabetes mellitus	10
Smoking habits	
Never smokers	18
Former smokers	25
Current smokers	7
Mean total plaque volume (range)	943.3±286.0 (458.5-2000.6) mm^3^
Mean LRNC volume (range)	96.5±147.44 (0-557.2) mm^3^
FC status	
Thick and intact	29
Thin and/or ruptured	21
IPH present	16
Mean ipsilateral WML	
volume at baseline (range)	6.52±8.17 (0.23-29.72) ml
Mean ipsilateral WML	
volume after one year (range)	6.97±8.88 (0.17-32.02) ml

**Figure 3:**
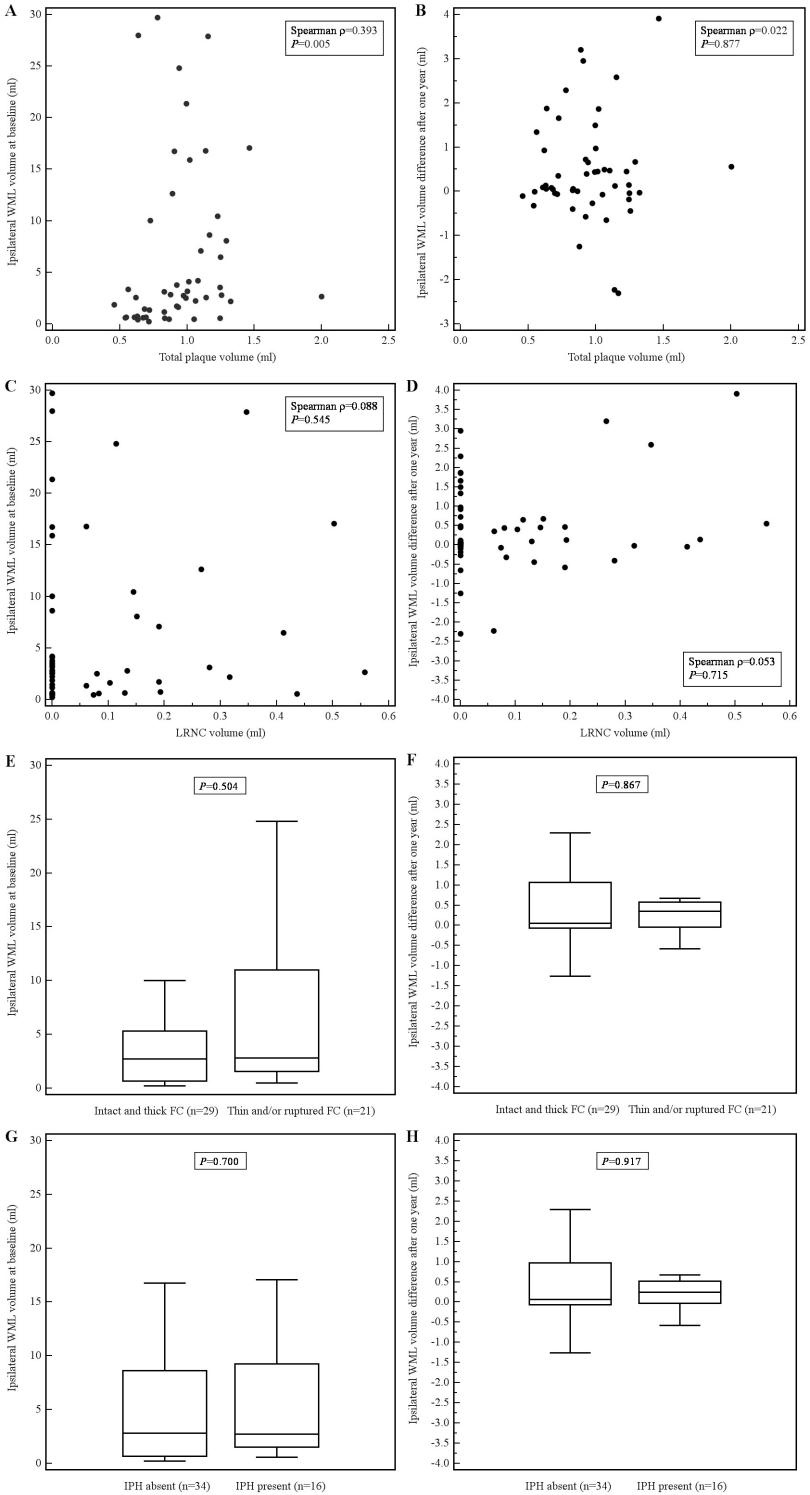
Scatter plots showing the relation between total plaque volume and ipsilateral WML volume at baseline (A) and ipsilateral WML volume difference after one year (B); and between LRNC volume and ipsilateral WML volume at baseline (C) and ipsilateral WML volume difference after one year (D). Box-and-Whisker plots showing the relation between fibrous cap status and WML volume at baseline (E) and WML volume difference after one year (F). Box-and-Whisker plots showing the relation between intraplaque hemorrhage and WML volume at baseline (G) and WML volume difference after one year (H).doi:10.1371/journal.pone.0103924.g003
